# Different characteristics of retinal damage between chronic hypertension and hypertensive retinopathy

**DOI:** 10.1038/s41598-022-23756-y

**Published:** 2022-11-07

**Authors:** Ki-Yup Nam, Min-Woo Lee, Ji-Ho Jun, Jae-Yun Sung, Jung-Yeul Kim

**Affiliations:** 1 Department of Ophthalmology, Chungnam National University Sejong Hospital, Sejong, Republic of Korea; 2 Department of Ophthalmology, Konyang University College of Medicine, 1643 Gwanjeo-Dong, Seo-Gu, Daejeon, Republic of Korea; 3 Department of Ophthalmology, Chungnam National University Hospital, #640 Daesa-Dong, Jung-Gu, Daejeon, 301-721 Republic of Korea; 4 1.0 Eye Hospital, Daejeon, Republic of Korea

**Keywords:** Diseases, Eye diseases, Retinal diseases

## Abstract

The purpose of this study was to identify how chronic hypertension (HTN) and hypertensive retinopathy (HTNR) have different effects on retinal damage including inner retinal thinning and microvasculature impairment. The subjects were divided into three groups: controls, HTN patients without HTNR (HTN group), and patients with relieved HTNR (HTNR group). The ganglion cell-inner plexiform layer (GC-IPL) thickness, vessel density (VD), and GC-IPL/VD ratio were compared among the groups. A total of 241 eyes were enrolled; 101 in the control group, 92 in the HTN group, and 48 in the HTNR group. The mean GC-IPL thicknesses were 83.5 ± 5.7, 82.1 ± 6.2, and 75.9 ± 10.7 μm in each group, respectively (*P* < 0.001). The VD was 20.5 ± 1.3, 19.6 ± 1.4, and 19.5 ± 1.6 mm^−1^ in each group, respectively (*P* = 0.001). The GC-IPL/VD ratio was 4.10 ± 0.33, 4.20 ± 0.40, and 3.88 ± 0.56 in each group, respectively (*P* < 0.001). In the HTNR group, HTN duration (B = 0.054, *P* = 0.013) and systolic blood pressure (SBP) (B = −0.012, *P* = 0.004) were significantly associated with the GC-IPL/VD ratio. In conclusion, inner retinal reduction and retinal microvasculature impairment were observed in patients with HTN and HTNR, and the GC-IPL/VD ratio of HTNR patients was significantly lower than that of HTN patients, indicating more prominent damage to the inner retina than microvasculature in HTNR patients. Additionally, the GC-IPL/VD ratio was significantly associated with SBP in HTNR patients, so more strict BP control is required in HTNR patients.

## Introduction

Hypertension (HTN), which causes cardiovascular, renal, and cerebrovascular diseases, can cause various damage to the retina^1,2^. Using optical coherence tomography (OCT), previous studies reported that HTN patients exhibited thinner ganglion cell-inner plexiform layer (GC-IPL) and peripapillary retinal nerve fiber layer (pRNFL) than normal controls^3–5^. With the development of optical coherence tomography angiography (OCTA), various damage of retinal microvasculature due to HTN has been reported. Sun et al.^6^ found that eyes of HTN patients exhibited reduced macular vessel density (VD) of the superficial and deep venous plexuses compared to controls. Shin et al.^7^ reported that the peripapillary VD and perfusion density (PD) were lower in patients with HTN for ≥ 10 years than normal controls. As such, chronic HTN causes both inner retinal reduction and impairment of retinal microvasculature.

Uncontrolled HTN can progress to hypertensive retinopathy (HTNR) showing narrowing of the retinal arteries, arteriovenous crossing, retinal microaneurysms and hemorrhages, hard exudates, and cotton-wool spots^8,9^. HTNR can leave damage to the retina even after it has been relieved. Lee et al.^4^ reported that the central macula, pRNFL, and GC-IPL were thinner in relieved HTNR patients than chronic HTN patients and normal controls. Peng et al.^3^ reported that reduction of retinal VD was observed in HTNR patients. As such, both chronic HTN and HTNR can induce inner retinal thinning and impairment of microvasculature. However, the mechanisms may differ; the chronic changes induced by HTN and acute damage caused by HTNR may affect the retina in different ways and to different extents.

In this study, we explored the different effects of chronic HTN and HTNR on retinal damage including inner retinal reduction and microvasculature impairment by analyzing macular VD, GC-IPL thickness, and GC-IPL/VD ratio in HTN and HTNR patients.

## Methods

This retrospective, cross-sectional study adhered to the tenets of the Declaration of Helsinki and was approved by the Institutional Review Board/Ethics Committee of Chungnam National University Hospital, Daejeon, Republic of Korea. The requirement for obtaining informed patient consent was waived due to the retrospective nature of the study. We reviewed the charts of patients who visited Chungnam National University Retinal Clinic from March 2018 to September 2021. HTN (clinical blood pressure, ≥ 140/90 mmHg; home blood pressure [BP], ≥ 135/85 mmHg) was diagnosed based on the Korean HTN treatment guidelines^10^. We investigated the detailed medical history, best-corrected visual acuity (BCVA), intraocular pressure measured using noncontact tonometry (Canon TX-20P, Canon Inc, Tokyo, Japan), spherical equivalent, and axial length of each patient. The BP of all patients was measured with the ophthalmic examination by an automated BP device in the retinal clinic. If the BP exceeded the normal range, it was measured again and the mean value was recorded.

The subjects were divided into three groups: controls, HTN patients without HTNR (HTN group), and patients diagnosed with grade 3 or 4 HTNR according to the Keith-Wagener-Barker classification system previously but who have relieved HTNR at the time of the study (HTNR group)^11^. The exclusion criteria were a history of systemic disease including diabetes, any other retinal disease other than HTNR, glaucoma, optic nerve disorder, any prior intraocular surgery except for cataract extraction, an axial length > 26 mm, a BCVA < 30/40, and an intraocular pressure > 21 mmHg. We also excluded patients with BP higher than 160/100 mmHg after treatment to analyze the retina with a stable state as in a previous study^3^. One eye was randomly selected if both eyes met the inclusion criteria.

### OCT and OCTA measurements

**Figure 1:**
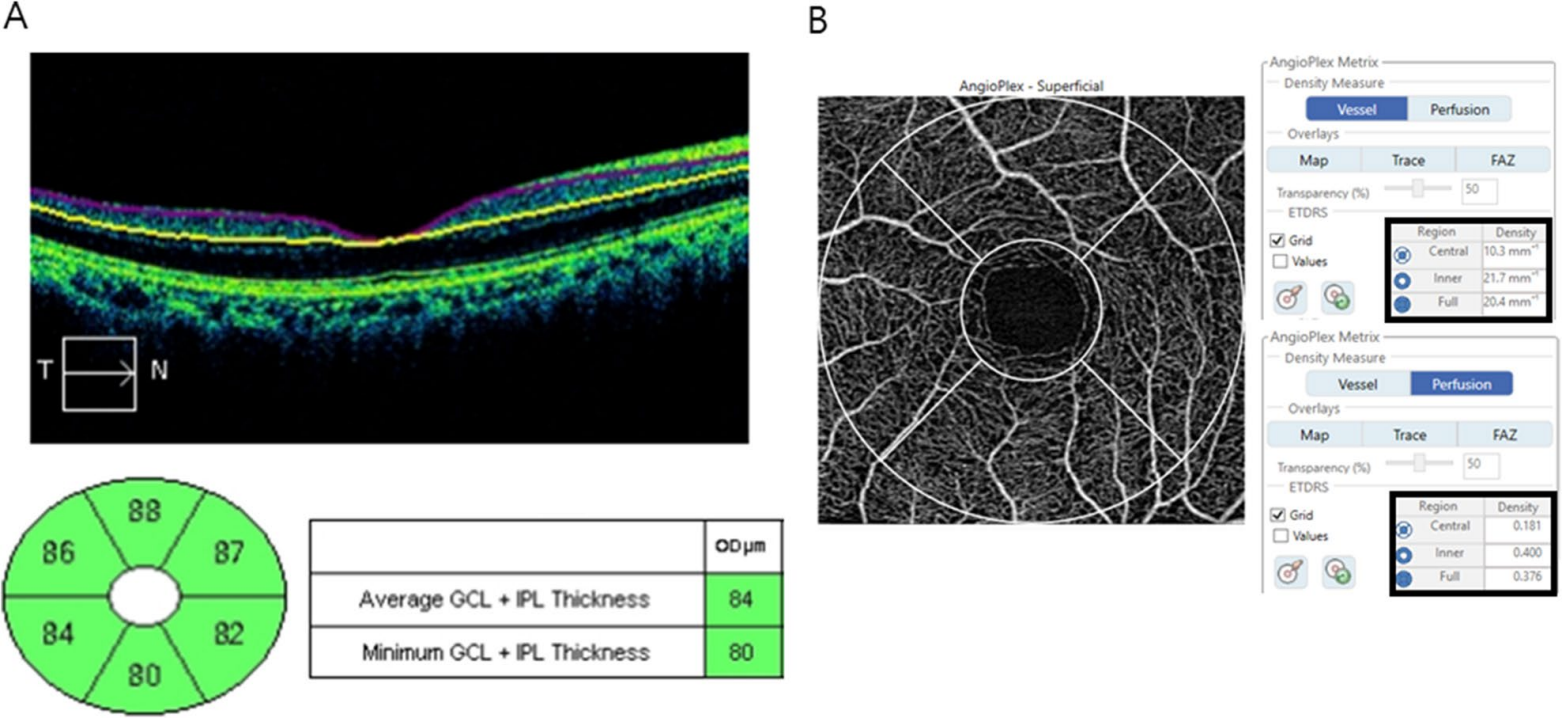
The thickness of the ganglion cell-inner plexiform layer (GC-IPL) using spectral-domain optical coherence tomography (**A**) and parameters of microvasculature using optical coherence tomography angiography (**B**).

Spectral domain OCT examinations were performed with the Cirrus HD-OCT 5000 instrument (Carl Zeiss Meditec, Dublin, CA) running the 512 × 128 macular cube scanning protocol. Central macular thickness was measured in the 1-mm diameter circle centered at the fovea, which is the central area of the Early Treatment Diabetic Retinopathy Study subfield. GC-IPL thickness was analyzed using an algorithm of the Ganglion Cell Analysis module (Fig. 1). The Ganglion Cell Analysis algorithm measured GC-IPL thickness automatically by identification of the outer boundaries of the macular RNFL and IPL using the 3D data of the macular cube scans. The average GC-IPL thickness and thicknesses of six sectors were analyzed. The pRNFL thickness was measured using an optic disc cube scan. The optic nerve head was brought to the center of the simultaneously scanned image, and a 200 × 200 resolution axial scan was performed over an area of 6 × 6 mm. The pRNFL thickness was measured at a diameter of 3.45 mm around the center of the optic disc. Images with low signal strength (< 7), decentration, or segmentation errors were excluded.

OCTA images were obtained using the Cirrus HD-OCT 5000 instrument running AngioPlex software (Carl Zeiss Meditec). AngioPlex gains high-resolution retinal microvascular images using a wavelength of 840 nm, taking 68,000 A-scan/s. An optical microangiography (OMAG) algorithm and retinal tracking technology ensure sensitivity and accuracy. We obtained 3 × 3-mm fovea-centered scans; all scans were analyzed using en face OCTA images generated automatically by the OMAG algorithm used in AngioPlex software. Each 3 × 3-mm scan was composed of a 1 mm center and sectors with four-quadrant, which were the inner circles of the Early Treatment of Diabetic Retinopathy Study (ETDRS). The central circle was 1 mm in diameter, the inner area included all four quadrant sectors, and the full area was the 3 mm-diameter inner circle of the ETDRS. The VD (the total length of perfused vasculature per unit area) and PD (the total area of perfused vasculature per unit area) of the superficial capillary plexus, which spanned from the internal limiting membrane to the IPL, were measured automatically by the software. The quality of all images was confirmed by two investigators (L.M.W., R.C.K.) individually; images with loss of fixation or foveal centration, segmentation errors, motion artifacts, and signal strength < 9 were excluded.

The GC-IPL/VD ratio was calculated by dividing the average GC-IPL thickness by the full area of VD.

### Statistical analysis

Demographic characteristics and ocular parameters were compared via one-way analysis of variance with post-hoc Bonferroni correction and the chi-squared test. An analysis of covariance was performed to control covarying factors. Patients with HTN and HTNR were classified into four subgroups according to the most commonly used regimens of antihypertensive treatment described in previous studies: (A) angiotensin-converting enzyme inhibitors and/or angiotensin-receptor blockers; (B) beta-blockers and/or calcium-channel blockers; (C) diuretics alone or combined with other medications; D) other combinations^12,13^. The subgroups divided according to the medication regimens were assigned to categorical factors for statistical analyses. In conditions with HTN or HTNR, both the inner retinal layer and microvasculature could be damaged, but differences in the severity of the two damages may exist between the groups. So we calculated the GC-IPL/VD ratio by dividing the average GC-IPL thickness by the full area of VD to intuitively understand the difference in the degree of damage between the inner retina and retinal microvasculature and compared it between the groups. Univariate and multivariate linear regression analyses, after adjusting for axial length, were performed to find the factors associated with the GC-IPL/VD ratio. All statistical analyses were performed using SPSS software (version 18.0; IBM Corp., Armonk, NY).

## Results

### Demographics

A total of 241 eyes were enrolled; 101 in the control group, 92 in the HTN group, and 48 in the HTNR group (Table 1). The average age was 50.9 ± 13.9, 52.3 ± 8.0, and 48.2 ± 9.9 years in the control, HTN group, and HTNR group, respectively, which was not significantly different (*P* = 0.081). The average BCVA was −0.04 ± 0.06, −0.01 ± 0.07, and 0.03 ± 0.14 in each group, respectively (*P* < 0.001). Sex, laterality, spherical equivalent, intraocular pressure, and axial length were not significantly different among the groups. HTN duration of HTN group and HTNR group was 7.4 ± 5.6 and 4.3 ± 3.9 years, respectively, which was significantly different (*P* < 0.001). The HTN medication was significantly different between the HTN group and HTNR group (*P* < 0.001); the number of subgroup A and B in the HTN group were larger than those in the HTNR group. The central macular thickness was 251.7 ± 19.1, 248.0 ± 19.2, and 243.3 ± 19.2 μm, respectively (*P* = 0.042), and pRNFL thickness 97.1 ± 9.3, 96.0 ± 9.1, and 91.9 ± 16.0 μm in each group, respectively (*P* = 0.025). In post-hoc analyses, the central macular thickness and pRNFL thickness of the HTNR group were significantly thinner than those of the control group (*P* = 0.039 and *P* = 0.022, respectively).

**Table 1:** Demographic and clinical characteristics.

	Control group (n = 101)	HTN group (n = 92)	HTNR group (n = 48)	*P* value
Age (mean ± SD, years)	50.9 ± 13.9	52.3 ± 8.0	48.2 ± 9.9	0.081
Sex (male, %)	49 (48.5)	38 (41.3)	26 (54.2)	0.320
Laterality (right, %)	53 (52.5)	53 (57.6)	25 (52.1)	0.828
BCVA (mean ± SD, logMAR)	−0.04 ± 0.06	−0.01 ± 0.07	0.03 ± 0.14	** < 0.001**
SE (mean ± SD, diopter)	−1.00 ± 2.38	−0.49 ± 2.49	−1.41 ± 2.49	0.091
IOP (mean ± SD, mmHg)	15.5 ± 2.9	15.2 ± 2.9	15.4 ± 3.3	0.831
Axial length (mean ± SD, mm)	24.2 ± 1.4	23.8 ± 1.3	24.1 ± 1.3	0.087
HTN duration (mean ± SD, years)	n/a	7.4 ± 5.6	4.3 ± 3.9	** < 0.001**
SBP (mean ± SD, mmHg)	122.7 ± 12.5	131.1 ± 11.3	134.9 ± 13.1	0.060
DBP (mean ± SD, mmHg)	79.2 ± 10.3	81.5 ± 9.9	84.1 ± 11.2	0.082
CMT (mean ± SD, μm)	251.7 ± 19.1	248.0 ± 19.2	243.3 ± 19.2	**0.042**
pRNFL (mean ± SD, μm)	97.1 ± 9.3	96.0 ± 9.1	91.9 ± 16.0	**0.025**

*BCVA* best-corrected visual acuity, *SE* spherical equivalent, *IOP* intraocular pressure, *HTN* hypertension, *SBP* systolic blood pressure, *DBP* diastolic blood pressure, *CMT* central macular thickness, *pRNFL* peripapillary retinal nerve fiber layer.

Values in boldface (*P* < 0.050) are statistically significant.

### GC-IPL thickness, macular microvasculature parameters, and GC-IPL/VD ratio

The average GC-IPL thicknesses were 83.5 ± 5.7, 82.1 ± 6.2, and 75.9 ± 10.7 μm in the control, HTN group, and HTNR group, respectively (*P* < 0.001 after adjustment for the BCVA) (Table 2). In post-hoc analyses, the GC-IPL thickness of the HTNR group was significantly thinner than that of the control and HTN groups (both *P* < 0.001), but the difference between the control group and HTN group was not statistically significant (*P* = 0.484). The comparison of sectoral thicknesses among groups showed similar results with that of the average thickness.

**Table 2:** Ganglion cell-inner plexform layer thickness and macular microvasculature parameters in each group.

	Control group	HTN group	HTNR group	*P* value
**GC-IPL**				
Average	83.5 ± 5.7	82.1 ± 6.2	75.9 ± 10.7	** < 0.001**
Superior	84.5 ± 6.6	82.8 ± 6.6	78.1 ± 12.4	**0.005**
Superotemporal	82.2 ± 6.0	81.7 ± 6.3	74.7 ± 10.5	** < 0.001**
Inferotemporal	83.6 ± 5.9	82.6 ± 6.5	73.6 ± 11.2	** < 0.001**
Inferior	81.1 ± 6.7	79.9 ± 6.5	73.6 ± 12.9	** < 0.001**
Inferonasal	83.6 ± 6.7	81.5 ± 7.3	76.1 ± 11.6	** < 0.001**
Superonasal	85.6 ± 6.6	84.1 ± 7.2	79.4 ± 13.8	**0.021**
**Microvasculature**				
Central VD	10.0 ± 2.6	8.7 ± 2.7	10.0 ± 2.7	**0.001**
Inner VD	21.8 ± 1.3	20.9 ± 1.3	20.7 ± 1.6	** < 0.001**
Full VD	20.5 ± 1.3	19.6 ± 1.4	19.5 ± 1.6	**0.001**
Central PD	17.6 ± 5.1	15.1 ± 4.9	17.6 ± 4.7	**0.001**
Inner PD	39.0 ± 2.2	37.8 ± 2.3	37.7 ± 2.7	**0.010**
Full PD	36.3 ± 3.0	35.3 ± 2.4	35.5 ± 2.7	0.078
GC-IPL/VD ratio	4.10 ± 0.33	4.20 ± 0.40	3.88 ± 0.56	** < 0.001**

*GC-IPL* ganglion cell-inner plexiform layer, *VD* vessel density, *PD* perfusion density.

*P* value after adjustment for covariants.

All values are expressed as the mean ± standard deviation (μm).

Values in boldface (*P* < 0.050) are statistically significant.

The full VD was 20.5 ± 1.3, 19.6 ± 1.4, and 19.5 ± 1.6 mm^-1^ in the control group, HTN group, and HTNR group, respectively (*P* = 0.001 after adjustment for the BCVA). In post-hoc analyses, the VD of the HTN and HTNR groups was significantly lower than that of the control group (*P* < 0.001 and *P* = 0.002), and the difference of VD between the HTN group and HTNR group was not statistically significant (*P* = 0.998). The inner VD was 21.8 ± 1.3, 20.9 ± 1.3, and 20.7 ± 1.6 mm^-1^ in each group, respectively (*P* < 0.001), and the VD of the HTN group and HTNR group was significantly lower than that of the control group (*P* = 0.001 and *P* < 0.001). The comparison of PD among groups showed similar results with that of VD.

**Figure 2:**
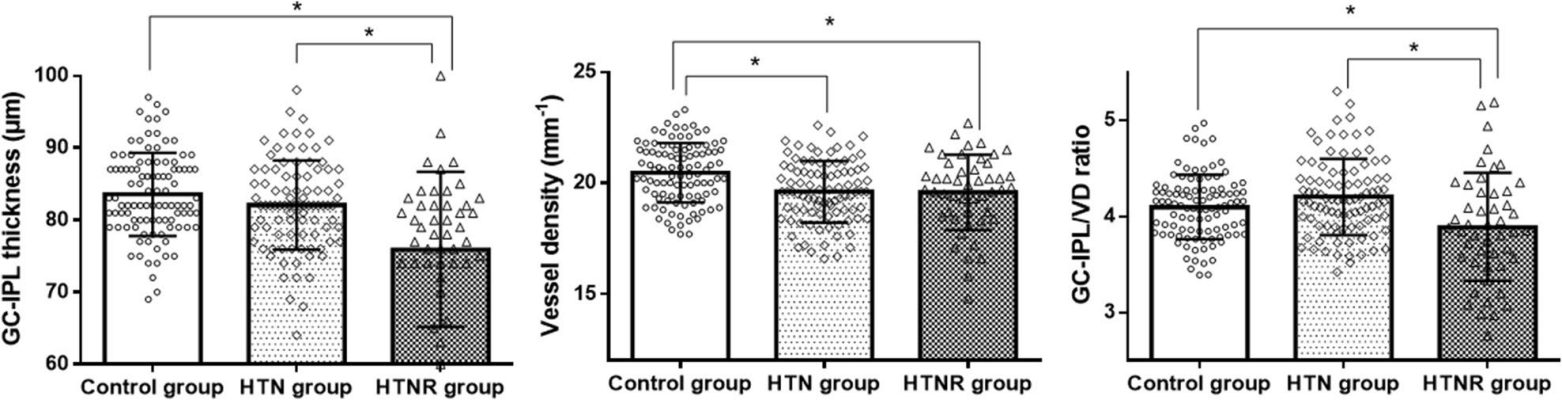
Bar graphs with scatter plots showing the average ganglion cell-inner plexiform layer thickness (GC-IPL), vessel density (VD), and GC-IPL/VD ratio in each group. *Statistically significant difference.

The GC-IPL/VD ratios were 4.10 ± 0.33, 4.20 ± 0.40, and 3.88 ± 0.56 in the control group, HTN group, and HTNR group, respectively (*P* < 0.001 after adjustment for BCVA). In post-hoc analysis, the ratio of the HTNR group was significantly lower than those of the control and HTN groups (*P* = 0.013 and *P* < 0.001), but the difference between the control group and HTN group was not statistically significant (*P* = 0.221) (Fig. 2).

### Univariate and multivariate linear regression analyses between various factors and GC-IPL/VD ratio

No factor was significantly associated with the GC-IPL/VD ratio in HTN patients (Table 3). In patients with relieved HTNR, the intraocular pressure (P = 0.008), HTN duration (*P* = 0.001), systolic BP (SBP) (*P* = 0.001), and diastolic BP (DBP) (P = 0.031) were significant factors associated with GC-IPL/VD in univariate analyses. We performed multivariate analyses using two models: Model 1 included the intraocular pressure, HTN duration, and SBP, and Model 2 included the intraocular pressure, HTN duration, and DBP as independent variables to avoid bias caused by multicollinearity. In Model 1, HTN duration (B = 0.054, *P* = 0.013) and SBP (B = −0.012, P = 0.004) showed significant result (Table 4). Whereas, only HTN duration (B = 0.065, *P* = 0.010) showed significant result in Model 2 (DBP; B = −0.005, *P* = 0.393).

**Table 3:** Univariate linear regression analyses for determining the factors associated with GC-IPL/VD ratio in patients with hypertension.

	B (95% CI)	P-value
Age	−0.007 (−0.017 to 0.004)	0.212
Sex	0.033 (−0.133 to 0.200)	0.693
Laterality	0.061 (−0.098 to 0.220)	0.447
BCVA	−0.064 (−1.221 to 1.093)	0.913
IOP	−0.001 (−0.029 to 0.026)	0.924
HTN duration	0.009 (−0.008 to 0.023)	0.199
SBP	−0.003 (−0.009 to 0.002)	0.209
DBP	−0.008 (−0.016 to 0.001)	0.082
HTN medication	0.054 (−0.030 to 0.139)	0.204
CMT	0.002 (−0.002 to 0.006)	0.340

*BCVA* best-corrected visual acuity, *IOP* intraocular pressure, *HTN* hypertension, *SBP* systolic pressure, *DB*P diastolic pressure, CMT central macular thickness.

**Table 4:** Univariate and multivariate linear regression analyses for determining the factors associated with GC-IPL/VD ratio in patients with hypertensive retinopathy.

	Univariate		Multivariate	
	B (95% CI)	*P* value	B (95% CI)	*P* value
Age	0.007 (−0.014 to 0.029)	0.500		
Sex	0.102 (−0.279 to 0.482)	0.591		
Laterality	−0.092 (−0.464 to 0.279)	0.617		
BCVA	−0.885 (−2.046 to 0.276)	0.131		
IOP	−0.077 (−0.133 to −0.021)	**0.008**	−0.015 (−0.071 to 0.041)	0.598
HTN duration	0.081 (0.035 to 0.127)	**0.001**	0.054 (0.012 to 0.096)	**0.013**
SBP	−0.012 (−0.019 to −0.006)	**0.001**	−0.012 (−0.020 to −0.004)	**0.004**
DBP	−0.011 (−0.022 to −0.001)	**0.031**		
HTN medication	−0.080 (−0.556 to 0.396)	0.733		
CMT	−0.002 (−0.011 to 0.008)	0.694		

*BCVA* best-corrected visual acuity, *IOP* intraocular pressure, *HTN* hypertension, *SBP* systolic pressure, *DBP* diastolic pressure, *CMT* central macular thickness.

Values in boldface (*P* < 0.050) are statistically significant.

## Discussion

Previous studies found a significant association between the retinal microvasculature and inner retinal layer thickness of the pRNFL and GC-IPL in HTN patients^7,14^. Atrophy of the inner retina may cause impairment of microvasculature, and impaired retinal perfusion can lead to reducing inner retinal layer thickness. However, the degree of damage caused by HTN could differ between the inner retinal structure and retinal microvasculature. In this study, we found that both HTN and HTNR patients exhibited thinner GC-IPL and lower VD and PD, and GC-IPL/VD ratio of HTNR patients was significantly lower than that of HTN patients, indicating more severe damage to inner retinal structure than microvasculature in HTNR. Additionally, this ratio was significantly associated with HTN duration and SBP in HTNR patients, whereas no factor showed a significant association in HTN patients.

Previous studies reported an inner retinal reduction in HTN patients. Sahin et al.^15^ reported that the RNFL thickness was reduced in HTN patients, which may be associated with atherosclerosis. Lee et al.^4^ also reported a significantly thinner GC-IPL in HTN patients than normal controls. In our study, the GC-IPL thickness of HTN patients tended to be thinner than that of controls, but statistical significance was not attained, possibly because of the relatively short HTN duration of HTN patients. Whereas, HTNR patients had a significantly thinner GC-IPL than normal controls and HTN patients even though their HTN duration was shorter than that of HTN patients. Autoregulation is an important blood flow regulatory mechanism in the retina and optic nerve head^16,17^. Chronic HTN can cause changes in arterioles such as vasospasm, arteriosclerosis, vasodilation, or vasoconstriction by angiotensin, which would interfere with autoregulation of the retina^18^. Besides these chronic changes, acute damage by high-grade HTNR, which can be the presenting sign of hypertensive emergency and an acute life-threatening condition resulting from markedly increased BP that leads to acute end-organ damage, may result in more severe impairment of the inner retina by additional reduced retinal and choroidal capillary perfusion during a hypertensive crisis^19^.

The impairment of retinal microvasculature could also occur in HTN patients. Lim et al.^14^ reported significantly decreased macular VD and PD in HTN patients. Sun et al.^6^ also found that systemic HTN was associated with the decreased retinal flow and increased foveal avascular zone area and perimeter in the deep vascular plexus. Our study showed significantly reduced VD and PD in patients with HTN, which was consistent with previous studies. Retinal neural damage seemed to occur along with macular capillary rarefaction in HTN patients, which is similar to the coincidence of diabetic retinal neurodegeneration and retinal microvasculature impairment seen in diabetes patients^20,21^. The inner retinal layer and retinal microvasculature would be connected closely by neurovascular coupling, and previous studies found a significant association between the inner retinal layer and retinal microvasculature in both HTN and diabetes^14,22,23^. Retinal atrophy may impair the microvasculature, and compromised retinal perfusion may reduce retinal thickness. Therefore, the inner retinal layer and microvasculature would interact with each other and be damaged to a similar extent in such conditions with chronic diseases, which may be supported by the result that the GC-IPL/VD ratio of the normal controls and HTN patients did not significantly differ.

Meanwhile, the GC-IPL/VD ratio of the HTNR group was significantly lower than those of the control and HTN groups. This result comes from the fact that the macular microvasculature was damaged similarly to the HTN group, while the inner retina was much more severely damaged in the HTNR group than the HTN group. This result was consistent with the previous study which showed a significant difference in GC-IPL thickness between HTN patients with retinopathy and HTN patients without retinopathy whereas macular VD of parafoveal superficial vascular plexus did not^3^. Damages by HTN crisis would be much more severe on the retinal neural tissue than on the retinal microvasculature. Such a low GC-IPL/VD ratio would suggest the possibility that there had been an HTN crisis in the past even though HTN patients did not show the typical characteristics of high-grade HTNR on their current retina. HTNR is a significant risk factor for stroke, death from cardiovascular disease, and renal impairment, and the level of BP causing grade 3 or 4 HTNR is sufficient to cause damage to multiple organ systems including the kidney, heart, and brain^8,24–26^. Therefore, physicians should consider the possibility of other end-organ damage in HTN patients with severe inner retinal thinning compared to an impairment of retinal microvasculature even though their retina does not have the typical characteristics of high-grade HTNR.

The HTN duration was positively associated with the GC-IPL/VD ratio in patients with HTNR, which implies that impairment of macular microvasculature is more prominent than retinal neural damage over time after diagnosis of HTN. This result would come from the time difference of damage between retinal microvasculature and the inner retinal layer. Whereas the inner retinal layer and microvasculature are damaged to similar extents in HTN patients, a plateau of inner retinal damage may be achieved after acute severe damage during HTN crisis and the microvasculature would be damaged continuously in HTNR patients. A prospective longitudinal study is needed to determine whether the GC-IPL/VD ratio of HTNR patients will eventually become similar to that of HTN patients over a long time.

The SBP was negatively associated with the GC-IPL/VD ratio in HTNR patients, which indicates more susceptible damage of the inner retinal layer to SBP than microvasculature. Gangwani et al.^18^ reported that a higher SBP was associated with a thinner RNFL in a Chinese hypertensive population. Jung et al.^27^ found inverse correlations between the inner retinal layer volumes and SBP as well as DBP. The retinal microvasculature is also known to be affected by BP in HTN patients. Chua et al.^28^ reported that persons with poorly controlled BP, higher SBP, and higher mean arterial pressure showed a sparse retinal capillary density in HTN patients. However, the VD of the macular SVP and DVP did not show a significant correlation with home BP monitoring in patients with essential HTN in another study^3^. The other previous study did not also show the significant correlation between mean arterial pressure and the VD of the superficial retinal layer in essential HTN^29^. As such, although it is controversial whether retinal microvasculature is directly affected by BP in HTN patients, it seems clear that the inner retinal layer would be more sensitively affected by BP than the microvasculature in HTNR patients judging from the result that the GC-IPL/VD ratio was significantly associated with SBP only in the HTNR group, not in the HTN group. Therefore, stricter BP control would help reduce inner retinal layer damage in HTNR patients.

Our study had several limitations. First, it had a retrospective design, which may have involved selection bias. Second, we could not totally eliminate the possibility that some patients who had recovered from previous HTNR in the past were enrolled in the HTN group. Third, we analyzed only the microvasculature of SVP, because this was automatically assessed by AngioPlex. However, analysis of the SVP is known to be more accurate than that of the deep vascular plexus because of the projection artifact^30^. Fourth, analyses of BP in this study were restricted. Because of missing data or irregular follow-up intervals between patients, we could not analyze the BP fluctuation or the BP before antihypertensive treatment but only the BP values at the visit. Prospective longitudinal studies are needed in the future. The strength of this study is that we included high signal strength OCTA images, which enhanced accuracy. Additionally, this is the first study to identify differences in retinal damage characteristics between chronic HTN and HTNR.

In conclusion, inner retinal reduction and retinal microvasculature impairment were observed in patients with HTN and HTNR, and the GC-IPL/VD ratio of the HTNR patients was significantly lower than that of the HTN patients, indicating more prominent damage to the inner retina than microvasculature in the HTNR patients. Therefore, physicians should consider the possibility of other end-organ damage in HTN patients exhibiting more severe inner retinal thinning compared to an impairment of retinal microvasculature even though their retina does not have the typical characteristics of high-grade HTNR. Additionally, the GC-IPL/VD ratio was significantly associated with SBP in HTNR patients, so stricter BP control is required in HTNR patients to minimize damage to the inner retinal layer.

## Data Availability

The datasets used and/or analyzed during the current study available from the corresponding author on reasonable request.
